# Ultrasound-guided perineural injection of the saphenous nerve in goat cadavers

**DOI:** 10.1186/s13620-024-00278-9

**Published:** 2024-07-31

**Authors:** Xavier Torruella, Antonella Puggioni, Bruno Santos, Pieter Brama, Vilhelmiina Huuskonen

**Affiliations:** School of Veterinary Medicine, UCD Veterinary Hospital, University College Dublin, Belfield, Dublin, D04 W6F6 Ireland

**Keywords:** Saphenous nerve, Nerve block, Ultrasound-guided, Locoregional, Local anaesthesia, Goat anaesthesia

## Abstract

**Background:**

Surgery of the goat stifle joint requires good perioperative analgesia, ideally without affecting motor function in the postoperative period.

The objective of this study was to describe an ultrasound-guided technique for saphenous nerve block in goats. Eleven fresh female goat cadavers from two different age groups were used: seven of them were four years old with a mean ± SD body weight of 65.9 ± 7.3 kg. Four animals were six months old and their mean ± SD body weight was 20.1 ± 3.1 kg. The cadavers were positioned in lateral recumbency with the limb to be blocked lowermost. A high-frequency linear transducer (6–12 MHz) was used to localise the interfascial plane between the sartorius and the vastus medialis muscles and to identify the saphenous nerve on the medial aspect of the thigh, caudal to the femur, at the level of the femoral triangle. In 22 pelvic limbs 0.1 mL/kg of methylene blue was injected around the saphenous nerve under ultrasound guidance, followed by gross anatomical dissection. The length of circumferentially stained nerve was measured, and the success rate of achieving at least 1 cm of staining is presented with a 95% confidence interval (CI).

**Results:**

Although not all saphenous nerves were sonographically identified, their boundaries were defined as cranial to the femoral artery, lateral to the sartorius muscle, and medial to the vastus medialis and rectus femoris muscles, within the perivascular fat. During anatomical dissection, the overall dye solution distribution was graded as complete in 17/22 limbs indicating a 77.3% success rate [95% CI (0.598, 0.948)], partial in 3/22 limbs and failed in 2/22 limbs.

**Conclusions:**

The success rate of this study indicates the feasibility of employing the ultrasound-guided technique to perform saphenous nerve block in goats. However, further in-vivo studies are recommended to assess the block's clinical efficacy before implementation on clinical patients.

## Background

Surgery of the hind limb is known to produce acute pain in humans and small animals, and if left untreated, subsequent peripheral and central sensitisation can lead to chronic or maladaptive pain and delayed healing [1–3]. Therefore, adequate perioperative pain management is important to ensure the welfare and wellbeing of the animals in our care. The provision of appropriate analgesia to food producing animals is a major challenge due to legal restrictions, and there are very few efficient licensed analgesic drugs available. However, local anaesthetic drugs such as lidocaine can be legally administered, and locoregional anaesthesia is known to be efficient in decreasing intraoperative analgesia requirements in humans [4] and both small and large animals [5–7]. Locoregional techniques can prolong the time between the end of surgery and the first systemic analgesic administration, and they can also reduce the recovery time and the time from end of surgery to the first food intake [4–7].

Epidural anaesthesia has been extensively studied in goats, sheep and small animals [7–9]. These studies have highlighted the efficacy of epidural administration of local anaesthetics alone, or in combination with other drugs, in achieving adequate perioperative analgesia for hind limb surgeries. However, in dogs, when the administration of local anaesthetics into the epidural space is compared to perineural administration, i.e., to a nerve block, the perineural administration has been shown to have a lower incidence of intraoperative complications such as hypotension [5]. There is, therefore, an interest to develop new, both ultrasound and nerve stimulator-guided nerve blocks. Waag et al. (2014) described an ultrasound-guided technique to block the sciatic and femoral nerves in sheep cadavers [10]. Adami et al. (2011) used a nerve stimulator-guided technique to anaesthetise the sciatic and femoral nerves in live goats undergoing arthrotomy, using three different concentrations of bupivacaine, and noted reduction in the intraoperative analgesia requirement. However, they observed a higher incidence of motor block in the groups that received 0.5% and 0.75% bupivacaine, which caused distress in the postoperative period characterised by anxiety and behavioural changes [11].

Ideally, to minimise distress in goats undergoing orthopaedic hind limb surgery, the locoregional technique should induce a sensory block without affecting motor function. Costa-Farré et al. (2011) described an ultrasound-guided technique to inject 2% lidocaine perineural to the saphenous nerve in live dogs, resulting in a complete sensory block of the dermatomes innervated by the saphenous nerve without causing a motor block [12].

The main objectives of the present study were twofold: 1. to describe the sonographic anatomy of the saphenous nerve injection site in goat cadavers, and 2. to report the success rate of ultrasound-guided methylene blue injections close to the saphenous nerve after anatomical dissection. Our hypothesis was that the ultrasound-guided technique would have at least an 80% success rate in circumferentially staining the saphenous nerves of goat cadavers.

## Materials and methods

An exemption from full ethical review was granted by the University College Dublin Animal Research Ethics Committee (AREC-E-21–48-Huuskonen). The study was conducted on two separate days, with 11 days in between, and 11 fresh female experimental goat cadavers from two different age groups were used. The first group comprised of seven four-year-old animals with a mean ± SD body weight of 66 ± 7 kg, while the second group included four six-month-old animals with a mean ± SD body weight of 20 ± 3 kg. All animals had been euthanised on the day of the study, approximately 3 to 4 h before the start, for reasons unrelated to this study.

### Part 1 (Anatomical study)

The first phase of the study involved a comprehensive exploration of the sonographic anatomy of the goat saphenous nerve in the targeted injection point and its associated structures in the femoral triangle. This investigation was conducted collaboratively with a European specialist in veterinary diagnostic imaging (AP).

**Fig. 1:**
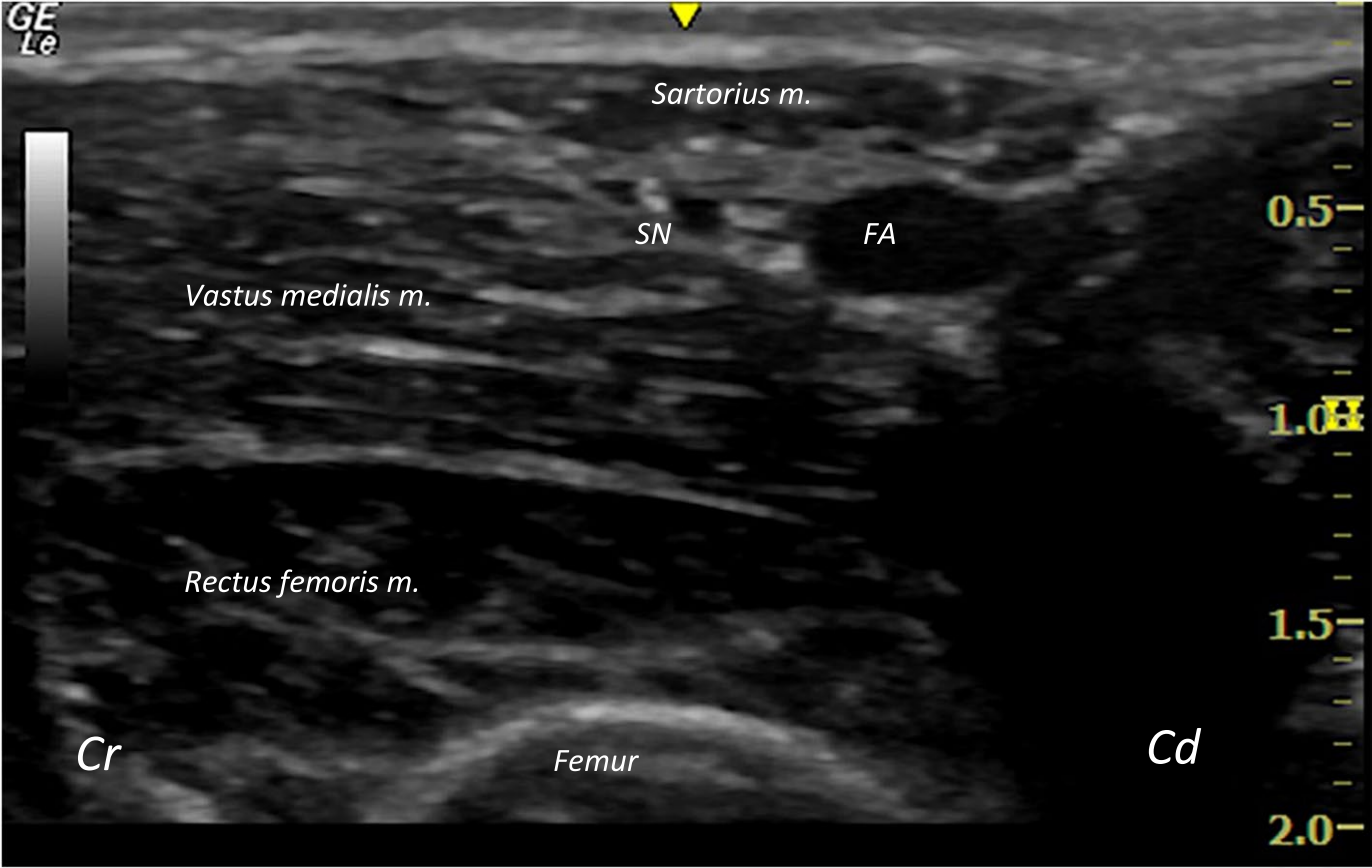
Ultrasound image of the medial aspect of the thigh of a goat cadaver. Ultrasound image of the medial aspect of the thigh before 1% methylene blue injection, showing the sonographic anatomy of the saphenous nerve injection point and the anatomical structures related to it. Cd: Caudal; Cr: Cranial; FA: Femoral artery; RF: Rectus femoris muscle; SM: Sartorius muscle; SN: Saphenous nerve

The goat cadavers’ inguinal and femoral regions were clipped, and alcohol was applied as a coupling medium to avoid the use of ultrasound gel. Two animals from the first group were scanned in detail using a portable ultrasound (US) unit^1^ 1 with a high-resolution linear transducer (6–12 MHz). The scans were performed by the first author (XT), who had previous experience in performing ultrasound-guided nerve blocks in other species, supervised by AP. Each medial aspect of the thigh region was scanned with the animal lying in lateral recumbency, right lateral for the right stifle and left lateral for the left stifle, to facilitate both scanning and injection. An assistant was holding the contralateral leg elevated. The US transducer was positioned on the medial aspect of the thigh, slightly caudal to the femur, in a perpendicular orientation compared to the long axis of the limb and with the marker facing cranially. Once the femoral artery was detected, the probe was slowly moved distally until the region of the saphenous nerve could be identified. As seen in the ultrasound screen, the skin and sartorius muscle were observed medially, the vastus medialis and rectus femoris muscle were observed laterally, and the saphenous nerve cranial to the femoral artery, forming a neurovascular bundle within the interfascial plane between the sartorius and vastus medialis muscles (Fig. 1). The saphenous nerve itself was seldomly clearly identified within the perivascular fat.

### Part 2 (US-guided perineural injection of the saphenous nerve followed by gross anatomical dissection)

**Fig. 2:**
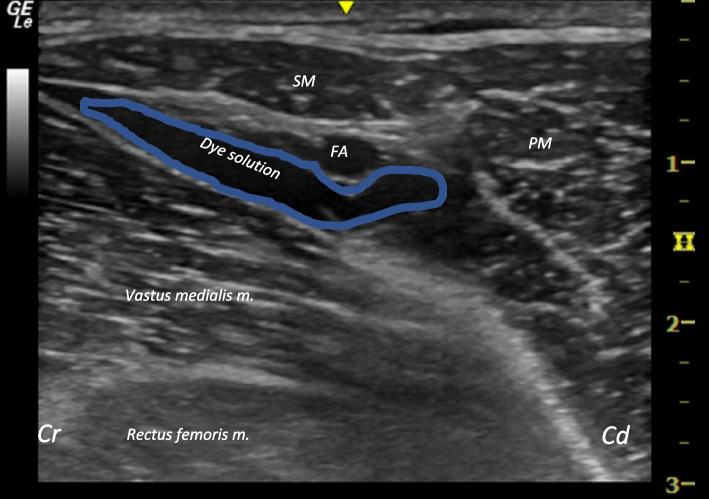
Ultrasound image of the saphenous nerve of a goat cadaver after dye injection. Ultrasound image of the medial aspect of the hind limb inguinal area after dye solution injection (1% methylene blue) showing the distribution of the dye between the sartorius muscle, vastus medialis muscle and the femoral artery. Cd: Caudal; Cr: Cranial; FA: Femoral artery; PM: Pectineus muscle; RF: Rectus femoris muscle; SM: Sartorius muscle; VM: Vastus medialis muscle

The second part consisted of the injection of 0.1 mL/kg of methylene blue solution^2^ 2 around the saphenous nerve, based on previous descriptions in dogs [12], followed by a gross anatomical dissection. Both inguinal areas and the medial thighs of the hind limbs were clipped, and the cadavers positioned in dorsal recumbency. This positioning facilitated scanning and avoided visual obstructions from the mammary glands and the contralateral limb, as noted in the first phase of the study. The operator was positioned on the right side of the cadaver, while the ultrasound machine was placed in front of the operator on the cadaver's left side. The probe was positioned perpendicular to the long axis of the limb, using an along-the-visual-axis technique. Keeping the femoral artery at the centre of the field of view, whether the saphenous nerve was identified or not, we targeted the femoral triangle at the point where the femoral vein and artery give rise to the saphenous vein and artery. A 22 G, 50 mm echogenic stimulating nerve block needle^3^ was inserted cranio-caudally at a 45-degree angle to the transducer; once the needle was recognised as a linear hyperechoic structure reaching the interfascial space, a small amount of methylene blue solution was injected as a trial. If the fluid appeared to be distending the interfascial space and compressing the surrounding vessels and muscles, the remaining amount of solution was injected, and the distribution of liquid observed under real-time imaging until a pocket of dye solution was observed between the femoral artery, the sartorius muscle and the vastus medialis muscle (i.e., within the femoral triangle, where the saphenous nerve should be located) (Fig. 2). Both hind limbs of all cadavers were injected. Our objective was to stain the saphenous nerve after the femoral nerve leaves the femoral triangle, and prior to the saphenous nerve branching to the stifle joint and the joint capsule.

The injection was followed by anatomical dissection performed with the help of a third year ECVS small animal surgery resident (BS) to assess the length of nerve exposure to the dye solution. For the anatomical dissection, a sharp skin incision was made parallel to the femoral bone, along the triangular space formed by the sartorius muscle cranially, the gracilis and pectineus muscles caudally, and the pelvic tendon medially and proximally. Blunt dissection of the muscles was performed using Metzenbaum scissors and Adson brown forceps. Identifying the femoral artery and vein was possible via superficial blunt dissection of the perivascular and perineural fat. Following the femoral artery and vein distally, the saphenous nerve, artery, and vein could be identified with retraction of the distal portions of the sartorius and gracilis muscles.

The length of circumferential nerve exposure to the methylene blue dye was measured in all 22 limbs using a 15 cm surgical disposable ruler^4^,^4^ and the success of the injection was graded as previously described by Portela et al. (2017): the staining was considered complete when the full circumference of the nerve was stained over a length of at least 1 cm (cm); partial when the stained area was less than 1 cm long or not circumferential; and failed when no exposure of the nerve with the dye solution was observed [13].

## Statistical analysis

The statistical software SPSS^5^ 5 was used for data analysis. The Shapiro–Wilk test was used to evaluate the normality of distribution of the following data: body weight within the age group; the total volume injected to each hind limb; and the length of the circumferentially stained nerve. Normally distributed data is expressed as mean ± standard deviation (SD), and the nonparametric data is expressed as median (range). The success rate of the injections was manually calculated, and the 95% confidence interval (CI) for the success rate was computed using the following commercial formula:

$$95\% interval= \widehat{P }\pm Z\ critical \sqrt{\widehat{P}} \left(1- \widehat{P}\right)/\text{ n},$$

where $\widehat{P}$ is the sample proportion, Z corresponds to the desired confidence level, and n is the sample size.

## Results

### Part 1 (anatomical study)

After scanning the first two cadavers, the ultrasound images obtained for the anatomical assessment were of good quality and all the anatomical landmarks related to the point of injection of the saphenous nerve were identified in the proximal and middle thirds of the femoral region. The femoral artery and vein were identified as hypoechoic round structures. The sartorius muscle displayed its characteristic triangular shape as it lay medially adjacent to the femoral artery, while the vastus medialis muscle occupied a lateral position. These three structures collectively defined the boundaries of the saphenous nerve, which when visible, was seen as a hyperechoic round structure situated cranially to the femoral artery, medially to the vastus medialis muscle, and laterally to the sartorius muscle (Fig. 1). When the transducer was slowly moved distally, it was observed how the femoral artery and vein gave origin to the saphenous artery and vein, respectively. Subsequently, these structures continued to extend distally in a more superficial location.

The femoral artery was identified in all 22 hind limbs. In 14 out of 22 hind limbs, in the four-year-old animals, the femoral artery was identified within an average depth of 1 cm from the skin. In 8 out of 22 hind limbs, in the 6-month-old animals, the femoral artery was identified within an average depth of 0.5 cm. Not all saphenous nerves were identified sonographically, and the number of nerves that were identified was not recorded.

### Part 2 (US-guided perineural injection of the saphenous nerve followed by gross anatomical dissection)

Ultrasound-guided injections of 0.1 mL/kg methylene blue were performed in both hind limbs of all 11 cadavers. In the four-year-old cadavers the mean ± SD volume injected was 6.6 ± 0.7 mL, while the six-month-old cadavers received 2.0 ± 0.3 mL.

During the anatomical dissection, all 22 saphenous nerves were identified. When following the origin of the saphenous artery and vein, branches of the saphenous nerve were observed to extend cranially toward the stifle joint (arising from the cutaneous branch of the saphenous nerve) at the same location where the saphenous nerve was detected sonographically: cranial to the femoral artery and vein before they gave origin to the saphenous artery and vein. Additionally, the distance between the saphenous nerve’s origin, its passage through the femoral triangle, and its branching to the stifle joint, seems to be subjectively shorter in goats when compared with the anatomy of the dog.

**Fig. 3:**
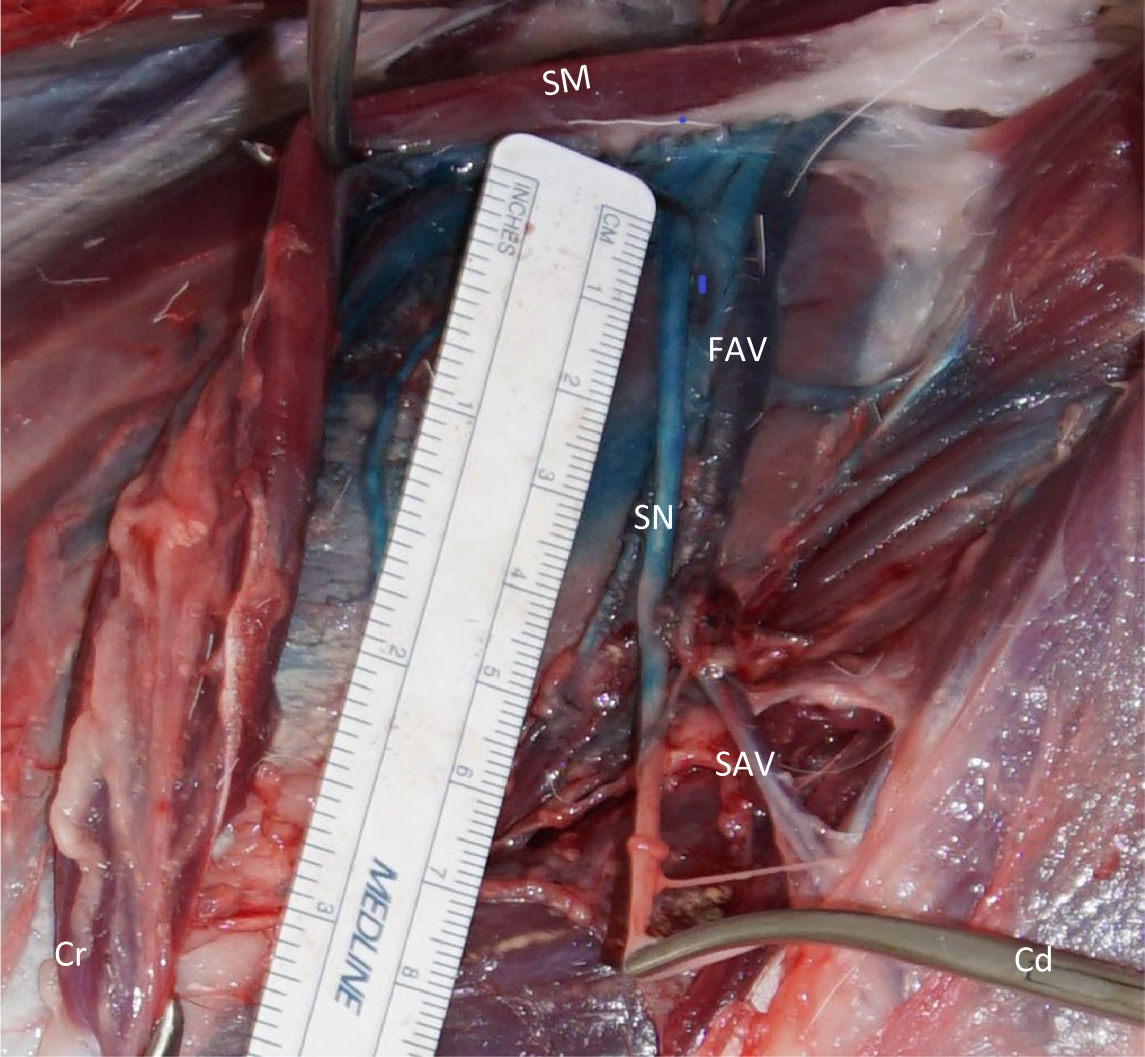
A hind limb inguinal area of a goat cadaver, dissected after dye injection. Dissected medial aspect hind limb (thigh area) showing the sartorius muscle which is dissected and retracted cranially to expose the saphenous nerve, and a ruler used for the assessment of the length of nerve exposure to the dye solution (1% methylene blue). Cd: Caudal; Cr: Cranial; FAV: Femoral artery and vein; SM: Sartorius muscle; SAV: Saphenous artery and vein; SN: Saphenous nerve

The length of circumferential nerve exposure to the methylene blue dye at this level of the saphenous nerve was measured in all 22 limbs, overall resulting in successful staining of the nerve in 77.3% or 17/22 limbs [95% CI (0.598, 0.948)]. The median (range) length of the nerve circumferentially exposed to the dye was 3 (1–4) cm. The staining was considered partial in 3/22 limbs and failed in 2/22 limbs [13] (Fig. 3). The success rate was higher in the smaller, younger cadavers than in the bigger, older cadavers (Table 1).

**Table 1:** Summary of the data

Number of animals	Age of cadaver (years)	Mean ± SD body weight (kg)	Number of nerves injected	Number of successfully stained nerves	Success rate (%) and 95% CI	Median (range) length of successful stain (cm)	Number of partially stained nerves	Length of partial stain (cm)
7	4	66 ± 7	14	10	71 (0.479, 0.949)	2.5 (1–4)	2	0.5 and 0.8
4	0.5	20 ± 3	8	7	88 (0.641, 1.0)	3 (1–4)	1	0.5

Summary of the data showing the number of goat cadavers used, the age and body weight of the cadavers, the success rate of the injections, and nerve exposure to dye. Eleven fresh goat cadavers from two different age groups received ultrasound-guided perineural injections around the saphenous nerve with 0.1 mL/kg of 1% methylene blue solution. After the injections, success rate was assessed through gross anatomical dissection. The success rate was judged based on the length of the circumferential staining of the nerve: the nerve staining was graded as complete if the circumferential staining was ≥ 1 cm, partial if < 1 cm, and failed if the nerve was not stained. The success rate is presented as percentage with its 95% confidence interval

## Discussion

In the present study, we describe the sonographic anatomy of the saphenous nerve injection point, the related anatomical landmarks, and the needle position before injection. We also report the success rate of ultrasound-guided perineural injection of 0.1 mL/kg methylene blue to the saphenous nerve (when sonographically identified) or cranial to the femoral artery (when the nerve was not sonographically identified).

As described in the literature, the femoral nerve of sheep, goats and dogs originates from the spinal nerves L4-L6, and then enters the psoas major muscle; before leaving the muscle, it gives rise to the saphenous nerve [10, 14, 15]. The saphenous nerve then becomes associated with the medial surface of the tensor fasciae latae muscle, and immediately divides into muscular and cutaneous branches. In dogs and goats, the muscular branch gives innervation to the cranial and caudal belly of the sartorius muscle, then continues distally next to the femoral artery and vein, and medial to the sartorius muscle [14, 15]. The cutaneous branch follows distally, cranial to the femoral artery, and gives innervation to the medial surface of the quadriceps muscle; it also sends branches to the skin of the middle and distal medial surfaces of the thigh. As it approaches the proximal part of the stifle joint, the saphenous nerve extends branches that innervate the medial aspect of the joint and its capsule [14, 15]. The approach used for injecting the dye solution in the present study facilitated staining the saphenous nerve before it branched to innervate the medial aspect of the stifle joint and joint capsule, as confirmed by gross anatomical dissection. In a live animal, the injection of local anaesthetic at this point should result in a sensory block of the stifle joint without significant motor blockade as has been described in dogs [12]; however, further clinical studies are required to assess its efficacy in goats.

The authors of the present study believe that the femoral artery and vein are the first and the easiest anatomical structures to be identified when looking for the target area in live goats, due to their characteristic hypoechoic round morphology and the femoral artery’s pulsatile behaviour. In the cadavers used in this study no pulsation was observed as expected, but the hypoechoic morphology was present, and the femoral vein was easily compressed with the transducer as opposed to the femoral artery. In dogs, the saphenous nerve is located on the midpoint of the medial aspect of the thigh and sonographically described as a discontinuous, hyperechoic, and oval structure, medial to the sartorius muscle and cranial to the femoral artery [12]. The point of injection in the present study (i.e., right before the femoral vein and artery give origin to the saphenous vein and artery) differs from the one described by Costa-Farré et al. (2011) in dogs [12], Nevertheless, in the present study, the sonographic anatomical characteristics were found to be comparable to those observed in dogs. Subsequent gross anatomical dissection of the cadavers used in the present study revealed that the anatomical location and characteristics of the nerves were also similar to those of dogs. In dogs, the saphenous nerve shares interfascial space with the femoral nerve in the proximal area of the femoral triangle. As the femoral nerve runs distally, it leaves the femoral triangle and enters the quadriceps femoris muscle [14]. Furthermore, in the present study, we observed that the distance between the saphenous nerve’s origin, its passage through the femoral triangle, and its branching to the stifle is subjectively shorter in goats than in dogs. This is why the targeted area in this study seems more proximal when compared to dog anatomy.

The goats used in the present study were euthanised 4–6 h prior to the injections. Due to the freshness of the cadavers, the ultrasound images obtained for the anatomical assessment were of good quality and all the anatomical landmarks related to the saphenous nerve were identified, although the saphenous nerve itself was not sonographically visualised in all hind limbs. The lack of visualisation could likely explain why only 17 out of 22 saphenous nerves were successfully stained. Waag et al. (2014) were also unable to sonographically identify six out of 26 femoral nerves in sheep cadavers; however, in their case it was due to tissue autolysis [10].

Unfortunately, we did not record how many ultrasound-guided injections in the present study were performed “blindly”; i.e., without visualising the nerve, nor how many injections were performed when the saphenous nerve was sonographically located. Recording the number of “blind” injections could have served to illustrate whether the effectiveness of the ultrasound-guided technique was compromised when the saphenous nerve was not sonographically identified prior to the injection and state the relationship between visibility and success rate of complete stain. Since the cadavers in the present study were very fresh, tissue autolysis did not interfere with nerve identification. Therefore, the lack of visualisation was most likely related to the learning curve of the operator (XT). The operator did not have a chance to thoroughly practise the technique before starting the data collection for this study, which likely had a negative influence on the number of correctly identified and subsequently completely stained nerves. This is supported by the fact that the success rate was lower with the first group of older animals, in contrast to the second, younger group, in which the success rate in achieving complete circumferential staining of the nerves was higher.

Even though we failed to confirm our hypothesis, the 77.3% success rate obtained in the present study could still be adequate provided that the clinical usefulness of this technique is validated in live goats. The efficacy of a local anaesthetic to block the neural conduction and action potential is dependent on the length of exposure of the nerve to the local anaesthetic, the concentration of the local anaesthetic, and the volume of the local anaesthetic [16, 17]. Raymond et al. (1989) defined the critical length of nerve exposure to the local anaesthetic as the length which decreases the amplitude of the compound action potential (CAP) by at least 50% [18]. When the sciatic nerves of frogs were experimentally exposed to 0.02% lidocaine hydrochloride (HCl) to assess the exposure length required to decrease the nerve’s ability to conduct an electrical impulse, Raymond et al. (1989) showed that the amplitude of the CAP started to decrease when the nerve was exposed to lidocaine over a length of at least 6 mm, and it exponentially decreased further when the exposure length was between 1 and 3 cm, reaching the critical length before 2 cm [18]. Furthermore, when they exposed the nerves to varied concentrations of lidocaine, they discovered that the length of exposure required for a successful block was significantly influenced by the concentration [18]. This effect was observed across a range of exposure lengths, extending from 6 mm to up to 35 mm, using concentrations of lidocaine HCl between 0.013% and 0.054%, suggesting that shorter lengths necessitated notably higher local anaesthetic concentrations to achieve a successful blockage of nerve impulses [18]. Thus, shorter exposure lengths could at least theoretically result in successful nerve impulse blockades if the local anaesthetic concentration is sufficient, while even substantial exposure lengths may result in incomplete impulse blockades if the concentration is insufficient.

Portela et al. (2017) developed a grading system for the assessment of the staining of nerves after injection of dye solution: grade 0 (failure) if the nerve is free of dye; grade 1 (partial) if the nerve is stained over less than 1 cm length or if the dye is not affecting the entire circumference of the nerve; and grade 2 (complete) if the entire circumference of the nerve is dyed for at least 1 cm of its length [13]. This grading system has since been used in a number of canine cadaveric locoregional technique studies to assess the technique’s ability to achieve the desired length of nerve exposure to the dye [19–22]. In the present study, in those three hind limbs in which the block was categorised as partial, the circumferential staining of the nerve ranged between 0.5 and 0.8 cm. However, it is possible that in live animals, similar “partially” successful blocks would provide sufficient perioperative antinociception if a high enough concentration of the local anaesthetic was employed.

In the present study, methylene blue dye solution was injected around and close to the saphenous nerve at a volume of 0.1 mL/kg. Costa-Farré et al. (2011) used a similar volume of local anaesthetic to perform an ultrasound-guided saphenous nerve block in five anaesthetised dogs, resulting in a successful sensory block with no motor block in all enrolled dogs [12]. The same volume was also injected perineural to the sciatic and femoral nerves in live goats with the help of a neurostimulator [11], resulting in a sensory and motor block of the dermatomes associated with each nerve. This outcome served as a measure to gauge the success of the injection. However, a smaller volume might be sufficient: when Waag et al. (2014) injected a total volume of 0.5 mL of methylene blue around the femoral nerve in sheep cadavers using an ultrasound-guided technique, the dye solution successfully stained the femoral nerve with an average circumferential exposure length of 3.7 cm [10]. Their results were roughly similar, although more successful, to the present study in which the 17 completely stained nerves had a median circumferential exposure length of 3 cm.

A limitation of the present study is that no power calculation was performed prior to starting the project. However, the number of goat cadavers available was limited to 11. Nevertheless, the sample size was considered adequate when compared to previous cadaveric studies describing novel ultrasound-guided locoregional techniques. For example, Waag et al. (2014) used 13 sheep cadavers to describe an ultrasound-guided injection of the femoral nerve [10]; Portela et al. (2019) used nine dog cadavers to describe an ultrasound-guided erector spinae plane block [19]; Garbin et al. (2020) used 12 dog cadavers for the description of the ultrasound-guided quadratum lumborum plane block [20]; and Costa-Farré et al. (2011) used five live dogs to describe an ultrasound-guided saphenous block [12].

## Conclusions

In conclusion, validated by gross anatomical dissection, the ultrasound-guided saphenous nerve block technique performed in the present study demonstrated an overall 77.3% success rate in achieving complete nerve staining in the targeted injection point. With some practice, the technique may potentially be used to perform perineural injections of the saphenous nerve in live goats. However, before it is used in clinical patients, further studies are necessary to assess the efficacy of this block and its ability to provide sufficient perioperative analgesia for goats undergoing stifle joint surgery.

## Data Availability

The data used are available from the corresponding author on reasonable request.

## Abbreviations

AREC: Animal Research Ethics Committee
AP: Antonella Puggioni
BS: Bruno Santos
CI: Confidence interval
ECVS: European College of Veterinary Surgeons
HCl: Hydrochloride
MHz: Megahertz
PB: Pieter Brama
US: Ultrasound
SD: Standard deviation
VH: Vilhelmiina Huuskonen
XT: Xavier Torruella

## Footnotes

1 Logiq™-e GE Healthcare, China.

2 Methylene blue 1% alcoholic, Solmedia Ltd, UK.

3 Stimuplex® Ultra 360® Needle B Braun, Germany.

4 Sterile disposable ruler, Medline, USA.

5 Version 27.0, IBM, USA.
